# Key Regulatory Differentially Expressed Genes in the Blood of Atrial Septal Defect Children Treated With Occlusion Devices

**DOI:** 10.3389/fgene.2021.790426

**Published:** 2021-12-08

**Authors:** Bo-Ning Li, Quan-Dong Tang, Yan-Lian Tan, Liang Yan, Ling Sun, Wei-Bing Guo, Ming-Yang Qian, Allen Chen, Ying-Jun Luo, Zhou-Xia Zheng, Zhi-Wei Zhang, Hong-Ling Jia, Cong Liu

**Affiliations:** ^1^ The Department of Cardiology, Shenzhen Children’s Hospital, Shenzhen, China; ^2^ Department of Pathophysiology, The Key Immunopathology Laboratory of Guangdong Province, Shantou University Medical College, Shantou, China; ^3^ Department of Medical Biochemistry and Molecular Biology, School of Medicine, Jinan University, Guangzhou, China; ^4^ Guangdong Cardiovascular Institute, Guangdong Provincial People’s Hospital, Guangdong Academy of Medical Sciences, Guangzhou, China; ^5^ The Department of Cardiology, Zhong Shan Affiliated Hospital of Xiamen University, Xiamen, China; ^6^ Guangzhou Mendel Genomics and Medical Technology Co., Guangzhou, China

**Keywords:** atrial septal defects, interventional closure, differentiated expressed genes, RNA-sequencing analysis, congenital heart defects

## Abstract

Atrial septal defects (ASDs) are the most common types of cardiac septal defects in congenital heart defects. In addition to traditional therapy, interventional closure has become the main treatment method. However, the molecular events and mechanisms underlying the repair progress by occlusion device remain unknown. In this study, we aimed to characterize differentially expressed genes (DEGs) in the blood of patients treated with occlusion devices (metal or poly-L-lactic acid devices) using RNA-sequencing, and further validated them by qRT-PCR analysis to finally determine the expression of key mediating genes after closure of ASD treatment. The result showed that total 1,045 genes and 1,523 genes were expressed differently with significance in metal and poly-L-lactic acid devices treatment, respectively. The 115 overlap genes from the different sub-analyses are illustrated. The similarities and differences in gene expression reflect that the body response process involved after interventional therapy for ASDs has both different parts that do not overlap and the same part that crosses. The same portion of body response regulatory genes are key regulatory genes expressed in the blood of patients with ASDs treated with closure devices. The gene ontology enrichment analysis showed that biological processes affected in metal device therapy are immune response with CXCR4 genes and poly-L-lactic acid device treatment, and the key pathways are nuclear-transcribed mRNA catabolic process and proteins targeting endoplasmic reticulum process with ribosomal proteins (such as RPS26). We confirmed that CXCR4, TOB1, and DDIT4 gene expression are significantly downregulated toward the pre-therapy level after the post-treatment in both therapy groups by qRT-PCR. Our study suggests that the potential role of CXCR4, DDIT4, and TOB1 may be key regulatory genes in the process of endothelialization in the repair progress of ASDs, providing molecular insights into this progress for future studies.

## Introduction

Atrial septal defects (ASDs) are the most common types of congenital heart defects (CHDs) and typically present with left to right shunts, which account for up to 10% and 40% of all CHDs, respectively (Penny and Vick, 2011; Rao and Harris, 2017). The patients with ASDs may exhibit poor growth and development, decreased activity tolerance, repeated respiratory infections, and hyperhidrosis, and they are accompanied by heart enlargement, increased pulmonary circulation pressure and resistance, heart failure, and atrial arrhythmia (Huang et al., 2013; Leppert et al., 2016; Karunanithi et al., 2017; Wu et al., 2018; Pillai et al., 2019). Surgery as the traditional method to treat ASDs has several disadvantages including large trauma, long recovery time and permanent scar left. It is more serious that residual septal defects are frequently associated with surgery-related side-effect complications, such as reoperation, infection, sternotomy scarring, and even death (Gaynor et al., 2001; Oses et al., 2010). To avoid those side effects of surgery, interventional closure has been developed to the main treatment to septal defects (Huang et al., 2013; Shimpo et al., 2013; Morray, 2019).

During the past four decades, several nondegradable types of occluders based on shape memory alloys have been used in clinical settings. Compared with surgery, interventional therapy has become the first choice for ASDs with the advantages of less trauma, less pain, no scar, short hospital stays, less complications, and no need for blood transfusion and extracorporeal circulation. With the recent development of occlusion devices and improved implantation techniques, the use of transcatheter closure of ASDs has increased over the years, considering that the permanent existence of foreign non-degradable materials *in vivo* can cause many potential complications in the long term (Luermans et al., 2010; Abaci et al., 2013). On the other hand, the use of biodegradable materials in the construction of occluders may overcome the drawbacks of metal devices (Shi et al., 2019). So, the research and development of biodegradable occluders has emerged as a crucial field for interventional treatment of ASDs. However, the main biological phenomena triggered after treatment with either degradable occluders or metal occluders are the same, including cardiac remodeling phenomena triggered by hemodynamic changes and biological responses induced by occluders. Because metal and biodegradable occluders are derived from different materials and have different structures, the phenomenon of cardiac remodeling after treatment behaves differently. The same point lies in the biological response of the body induced by the occluder. In general, the occlusion device is used to provide a temporary scaffold for tissue endothelialization (Tang et al., 2018). Some studies revealed that endothelialization is related to cell proliferation, cell migration, and cell junction (Bazzoni and Dejana, 2004; Dejana 2004). The previous studies showed that normal expression of genes encoding transcription factors, cell signaling molecules, and structural proteins are important for heart development (Williams et al., 2019). It was also reported that both metal and biodegradable occluders are beneficial to endothelial cell coverage by histological and electron microscopic examinations (Li et al., 2019). However, whether the occlusion device affects ASD repair by regulating the expression of key genes remains unclear.

RNA-sequencing (RNA-Seq) is a useful method to explore the molecular events in many different samples, including blood, cells, and tissues. In this study, we performed differentially expressed genes (DEGs) analysis on RNA-sequencing data in blood samples of patients with the occlusion device (metal or biodegradable device) post-treatment group compared to the pre-therapy group. Combining with the expression level validation of DEGs by quantitative real-time PCR, our study aims to discover DEGs and overlap genes in ASDs, to illustrate the potential role of specific overlap genes and its function on biological processes.

## Materials and Methods

### Preparation of Samples

Between January 2019 and December 2019, pediatric patients undergoing closure of secundum ASD with either metal occluder or PLLA occluder in our hospital were included in this study. Indications for ASD closure were as follows: an ASD ≥5 mm and ≤30 mm in diameter, with sufficient rims of atrial tissue (superior to the coronary sinus, superior/inferior vena cava, and pulmonary vein by 5 mm and superior to the mitral valve by 7 mm), signs of right ventricular volume overload, and/or evidence of significant left-to-right shunting (Qp:Qs ≥ 1.5:1). Patients with other congenital or significant cardiac defects, history of ASD repair, metal implant, or PLLA implant were excluded from the study. The study was approved by the Committee on the Ethics of Shenzhen Children’s Hospital (202000903), and written informed consent was obtained from all guardians. The samples used for RNA-sequencing analysis were collected from Shenzhen Children’s Hospital, including two patient samples before and after metal device therapy (Cera™, Lifetech Scientific, Shenzhen, China), three patient samples before and after poly-L-lactic acid (PLLA) device therapy (Absnow™**,** Lifetech Scientific, Shenzhen, China), two patient samples before metal device therapy and one patient sample after PLLA device therapy, and four samples from healthy volunteers; the basic clinical characteristics of these children are listed in Table 1A. For qRT-PCR, the blood sample of treatment groups was randomly selected, including 11 samples before and after PLLA device therapy, 10 samples before and after metal device therapy, and 8 healthy people as the control group. The basic characteristics of the children for qRT-PCR analysis are listed in Table 1B. All patients with ASDs underwent interventional therapy and samples were collected that day before and 30 days after the intervention.

**TABLE 1A:** Basic characteristics of the patient samples of atrial septal defects used for RNA-sequencing analysis.

Samples source	Cases	Male/Female	Before/After therapy	Before/After therapy	Age (months)	Weight (kg)	Sptal defect (mm)	Qp/Qs	Occluder (mm)
PLLA device therapy patients	1	F	B	A	56	17.4	9	2.1	12
	2	F	B	A	68	14.2	9	1.9	14
	3	F	B	A	52	17.3	8	2.0	16
	4	M	—	A	13	10.3	6	1.8	12
Metal device therapy patients	5	F	B	A	10	9.3	7	1.9	12
	6	M	B	A	19	11.5	10	2.3	14
	7	F	B	—	29	13	8	1.7	12
	8	M	B	—	56	19	6	1.6	10
Healthy volunteers	9	M	—	—	11	8	—	—	—
	10	F	—	—	13	11	—	—	—
	11	F	—	—	19	12.2	—	—	—
	12	F	—	—	16	10.2	—	—	—

PLLA: poly-L-lactic acid; Qp/Qs: Pulmonary-to-Systemic-Blood-Flow Ratio.

**TABLE 1B:** Basic characteristics of the patient samples of atrial septal defects used for qRT-PCR.

Samples source	Cases	Male/Female	Age (months)	Weight (kg)	Sptal defect (mm)	Qp/Qs	Occluder (mm)
PLLA device therapy patients	*N* = 11	4/7	34.5 ± 20.2	12.8 ± 4.6	13.5 ± 5.0	2.1 ± 0.4	18.0 ± 5.4
Metal device therapy patients	*N* = 10	4/6	29.1 ± 21.1	12.9 ± 3.9	12.1 ± 0.8	1.9 ± 0.3	16.0 ± 1.5
Healthy volunteers	*N* = 8	2/6	24.1 ± 15.1	12.0 ± 3.0	—	—	—

PLLA: poly-L-lactic acid; Qp/Qs: Pulmonary-to-Systemic-Blood-Flow Ratio.

### RNA Extraction

The RNA was extracted from whole blood sample following the Trizol reagent manual (Invitrogen Life Technologies, Carlsbad, CA). In brief, 5 ml of Trizol reagent was added to 1 ml of whole blood sample for 10 min on ice, and then RNA was precipitated in 1:1 isopropanol/Trizol (v/v) and 1 μl of glycogen at −20°C overnight followed by use for mRNA-sequencing.

### Library Preparation

cDNA library preparation: total RNA (200 ng) was used to prepare cDNA libraries using the NEBNext Ultra RNA library prep kit for Illumina (New England Biolabs) following the manufacturer’s protocol. Quality and integrity of the tagged libraries were initially assessed with the HT DNA HiSens Reagent kit (Perkin Elmer) using a LabChip GX bioanalyzer (Caliper Life Sciences/Perkin Elmer). Tagged libraries were then sized and quantitated in duplicate (Agilent TapeStation system) using D1000 ScreenTape and reagents (Agilent). Sequencing was performed as PE150 on an Illumina NovaSeq 6000 sequencer. The high-quality reads that passed the Illumina filter were subjected to the subsequent bioinformatics analysis.

### Transcriptome Profiling

Adapters and low-quality bases with the sequencing reads for each sample were preprocessed by fastp (https://academic.oup.com/bioinformatics/article/34/17/i884/5093234) with a default setting. Filtered reads were mapped to the latest version of human genome (*Homo sapiens*, GRCh38) by STAR (https://doi.org/10.1093/bioinformatics/bts635) aligner with parameters: --outSAMtype BAM SortedByCoordinate, and the mapping results were summarized into a gene expression matrix using featureCounts v1.6 (https://doi.org/10.1093/bioinformatics/btt656). Output data were then processed with customized R scripts.

### Differential Expression Genes (DEGs) Analysis

Gene-level differential expression was analyzed using DESeq (https://doi.org/10.1089/omi.2011.0118) for the metal or poly-L-lactic acid device sample group, respectively. The ASDs pre-therapy or post-treatment were specified as the experimental design. Benjamini and Hochberg *p*-value adjustment methods were used for multiple comparisons. Parameter alpha (significance cutoff) was set to 0.1 and lfcThreadshold (log2 fold change threshold) was set to 0 following the best practice of DESeq pipeline. Genes with an absolute fold change (FC) greater two and a *p*-value less than 0.05 were selected for the downstream analysis.

### Gene Ontology Enrichment Analysis

DEGs were annotated by pre-defined terminologies such as GO analysis, and over-representation analysis (ORA) was performed by clusterProfiler (https://doi.org/10.1089/omi.2011.0118).

### Weighted Gene Co-Expression Network Analysis

Weighted Gene Co-Expression Network Analysis (WGCNA) was carried out to evaluate the correlation between genes and to classify highly correlated genes into the same module. The data submitted to the WGCNA R package (https://doi.org/10.1186/1471-2105-9-559) was firstly processed by differential expression analysis to filter out irrelevant information. The data submitted to WGCNA R package was firstly processed by Variance Stabilizing Transformation (VST) algorithm. The topological overlap measure (TOM) was employed to identify modules of highly co-expressed genes, and genes with high absolute correlations were clustered into the same modules by cutting the dendrogram into branches. The only number of genes that exceed 30 will be defined as a module. Then, pairwise correlations between gene modules and clinical datasets were calculated. Modules with higher correlation will be merged (*r* < 0.25); each module was assigned to different colors for visualization.

### Protein–Protein Interactions Analysis

PPIs are physical contacts of high specificity established between two or more protein molecules as a result of biochemical events steered by interactions that include electrostatic forces, hydrogen bonding, and the hydrophobic effect. PPI with known disease genes have been used to find new disease genes by identifying key core genes. We derived core genes by network connection scores to describe the module elements, including core and ring components. Functions of core genes were highly correlated with those of essential genes in the same modules.

### Quantitative Reverse Transcriptase-Polymerase Chain Reaction Analysis

Total RNA was extracted with Trizol according to the manufacturer’s instructions. Unique genomic DNA remover is combined with EasyScript^®^ First-Strand cDNA Synthesis SuperMix to achieve simultaneous genomic DNA removal and cDNA synthesis. The cDNA levels were measured by SYBR Green in real-time PCR using the LightCycler. The housekeeping gene GAPDH was used as normalized in each individual sample and the 2^−ΔΔCt^ method was used to quantify relative expression changes. The sequences of specific primers used for qRT-PCR assays in this study are listed in Supplementary Table S1.

### Statistical Analysis

Statistical significance was performed using Student’s *t*-test and *p* < 0.05 was considered statistically significant.

## Results

### The Expression Profile Diverse Before and After the Occlusion Device Therapy

**FIGURE 1:**
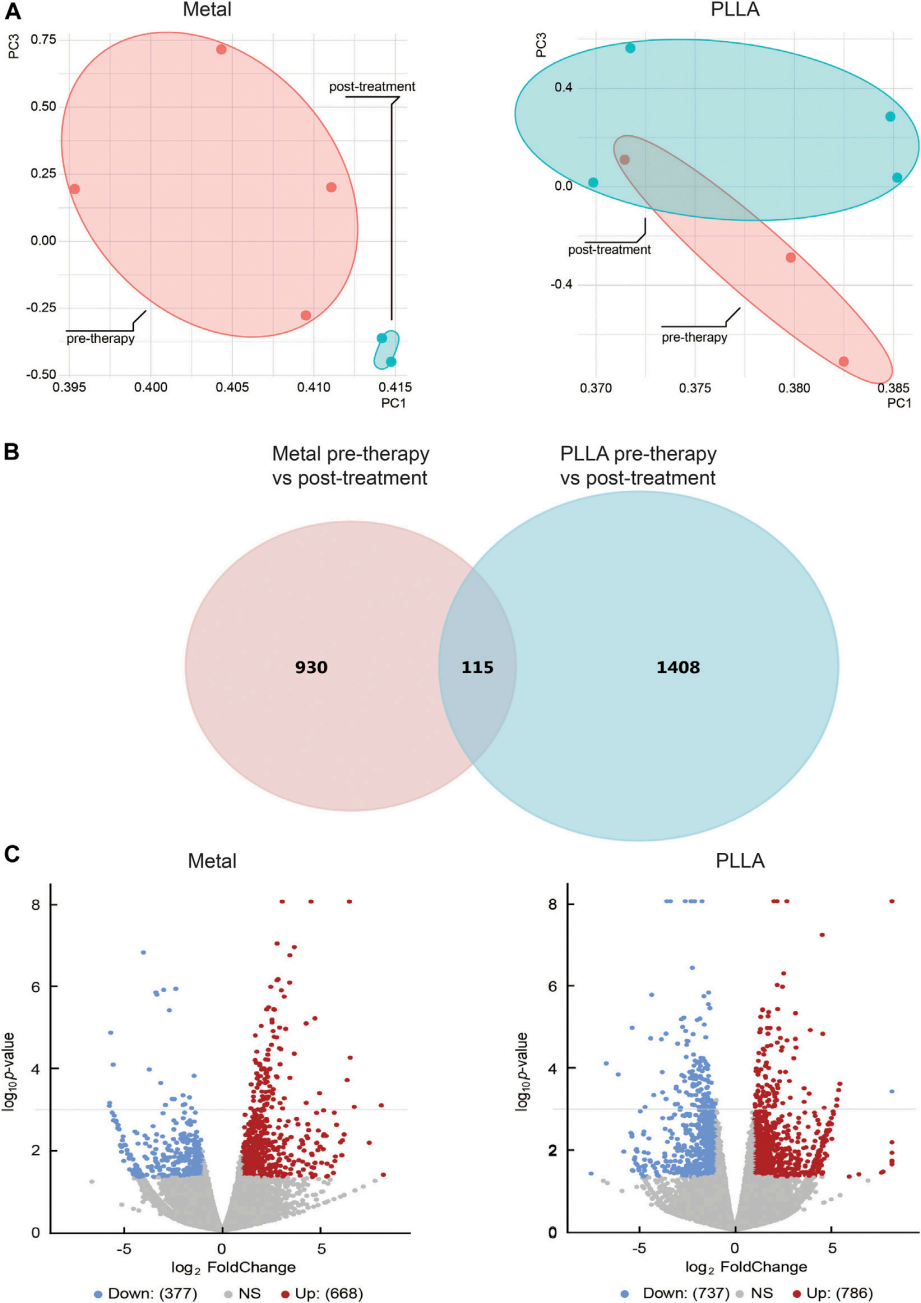
The differential expression genes (DEGs) analysis of atrial septal defects patients before and after metal and PLLA device therapy. **(A)** Samples can be distinguished by principal component analysis (PCA). **(B)** Venn diagram showing the genes identified in PLLA and metal device after and before therapy. **(C)** Significantly changed genes were discovered from differentially expression analysis. Gene with a *p*-value less than 0.05 and an absolute fold change greater than 2 is considered as a significantly changed gene. In each panel, the blue dots represented downregulated genes and the red dots represented upregulated genes.

**FIGURE 2:**
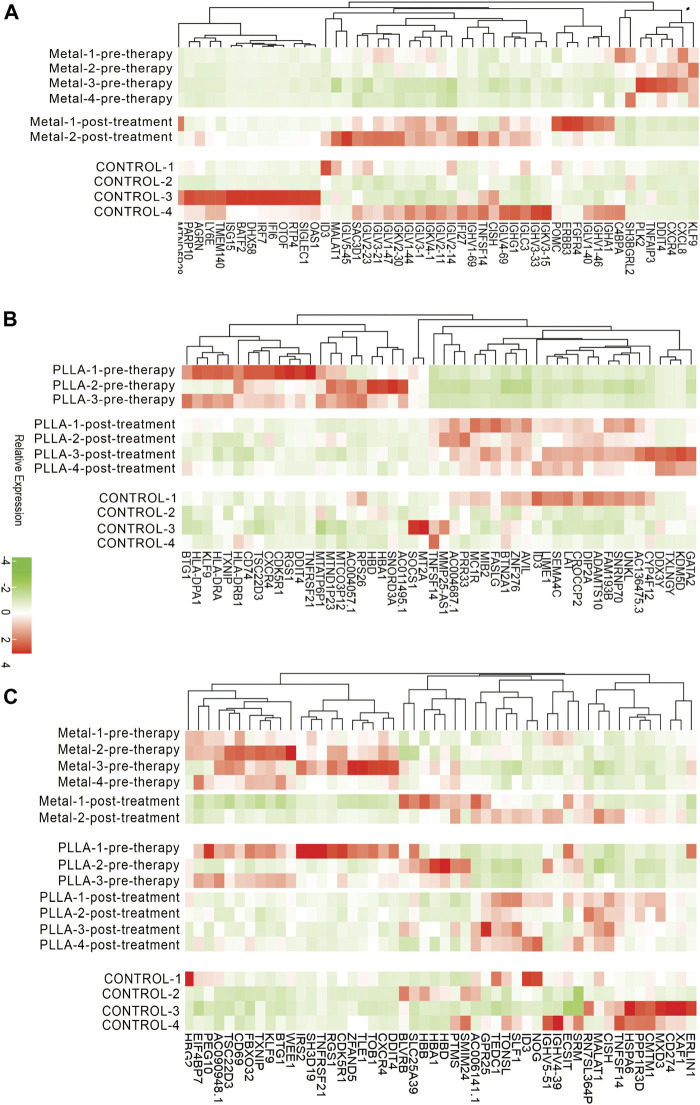
Expression levels of DEGs from metal and PLLA device groups. **(A)** Color key of the heatmap shows the relative expression level of DEGs in metal device pre-therapy group vs. post-treatment group. **(B)** Heatmap showing DEGs in PLLA device pre-therapy group vs. post-treatment group. **(C)** Heatmap of overlap DEGs between metal and PLLA device groups.

Previous studies showed that the occlusion device is used to provide a temporary scaffold for tissue endothelialization,15 but whether it plays a role in biological processes is unclear. Therefore, we explore the effects of the occlusion device on biological processes by RNA-Seq. The DEGs analysis was performed for ASDs cases and healthy control. Principal component analysis and inspection of the first two principal components illustrate the presence of four groups of samples (Figure 1A). DEGs were tested utilizing two strategies. Firstly, the occlusion device post-treatment pools were compared against the occlusion device pre-therapy pools. Secondly, the overlap genes of DEGs between PLLA and metal device post-treatment and pre-therapy pools were analyzed. From the sub-analyses, we obtained 1,523 genes and 1,045 genes that were statistically significantly differently expressed between the occlusion device post- and pre-treatment (Figure 1B). The overlap genes in the results from the different sub-analyses are illustrated, with 115 genes differently expressed (Figure 1B). The distribution of DEGs between the metal device post-treatment and pre-therapy or the PLLA device post-treatment and pre-therapy is shown in a volcano plot, respectively (Figure 1C). Among these genes, 337 genes were downregulated (blue dots) and 668 genes were upregulated (red dots) in metal device post-treatment vs. pre-therapy. Compared to the PLLA device pre-therapy, 737 genes were downregulated (blue dots) and 786 genes were upregulated (red dots) in PLLA device post-treatment (Figure 1C). Overlap of the top 50 DEGs in metal device groups and the top 50 PLLA device groups is shown in Figures 2A–C. In addition, Supplementary Tables S2–4 show the DEGs of metal device groups, PLLA device groups, and overlap genes by the occlusion device post-treatment vs. pre-therapy, respectively.

### Pathway and Functional Enrichment Analysis

**FIGURE 3:**
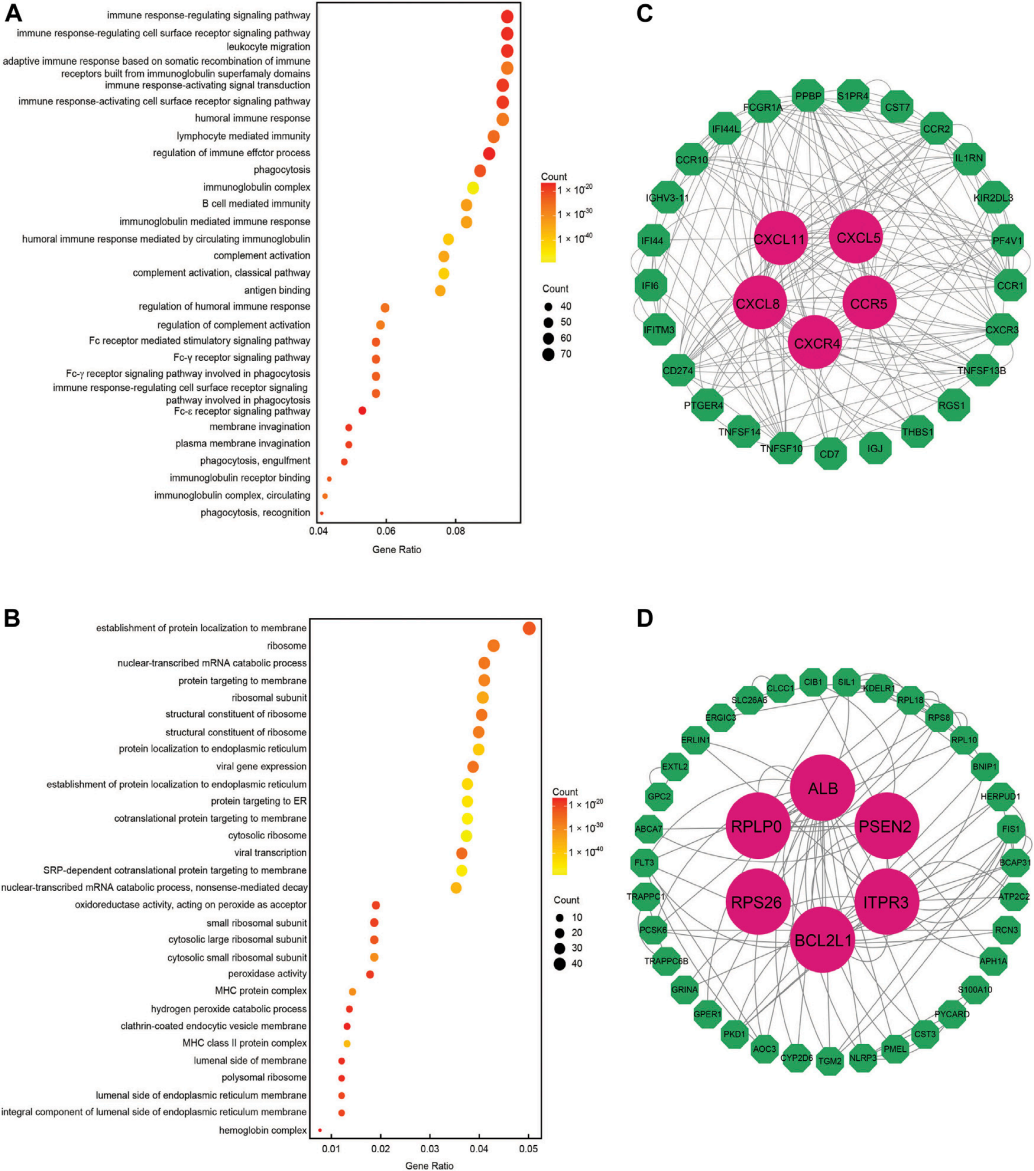
DEGs are significantly enriched in multiple functional groups. The position of each dot represents the ratio of gene number for each GO term. The absolute number of gene and significant level are annotated by size and color, respectively. **(A)** Significant GO terms for DEGs between the metal device post-treatment group and pre-therapy. **(B)** Significant GO terms for DEGs between the PLLA device post-treatment group and pre-therapy. **(C)** Protein–protein interactions (PPIs) for the metal device therapy group. The CXCR4 gene is the core gene for the immunity response pathway. **(D)** PPI analysis for the PLLA device therapy group. The RPS26 gene is the core gene for the proteins targeting the endoplasmic reticulum process pathway.

DEGs that the occlusion device treatment induced were analyzed in the above results. We subsequently compiled a list of the most frequently altered linked genes (including upregulated and downregulated genes), prior to analyzing this gene list using the GO tools in clusterProfiler (https://doi.org/10.1089/omi.2011.0118). Figure 3 summarizes the most significantly overrepresented GO terms in the biological process category and also PPI core gene analysis in metal and PLLA device therapy, respectively. We found that the following processes were affected by the occlusion device treatment: DEGs in the metal device group were enriched in immune response-regulating signaling pathway, immune response-regulating cell surface receptor, leukocyte migration, immune response-activating signal transduction, and immune response-activating cell surface receptor (Figure 3A). DEGs in the PLLA device group were most highly enriched for the GO terms establishment of proteins localization to membrane, nuclear-transcribed mRNA catabolic process, proteins targeting the membrane, proteins targeting the endoplasmic reticulum process, and establishment of protein localization to endoplasmic reticulum (Figure 3B). The biological processes identified in this analysis are likely to contribute to the pathobiology of the occlusion device treatment. These results suggest that mechanisms of development and remodeling of ASDs might be different in metal or PLLA device treatment. PPI analysis shows the core and ring genes in the immune response pathway and the CXCR4 are the core genes identified in the metal device group (Figure 3C), and the key pathways are nuclear-transcribed mRNA catabolic process and proteins targeting the endoplasmic reticulum process with ribosomal proteins (such as RPS26) in the PLLA device group (Figure 3D).

### Weighted Gene Co-Expression Network Analysis of Differentially Expressed Genes

**FIGURE 4:**
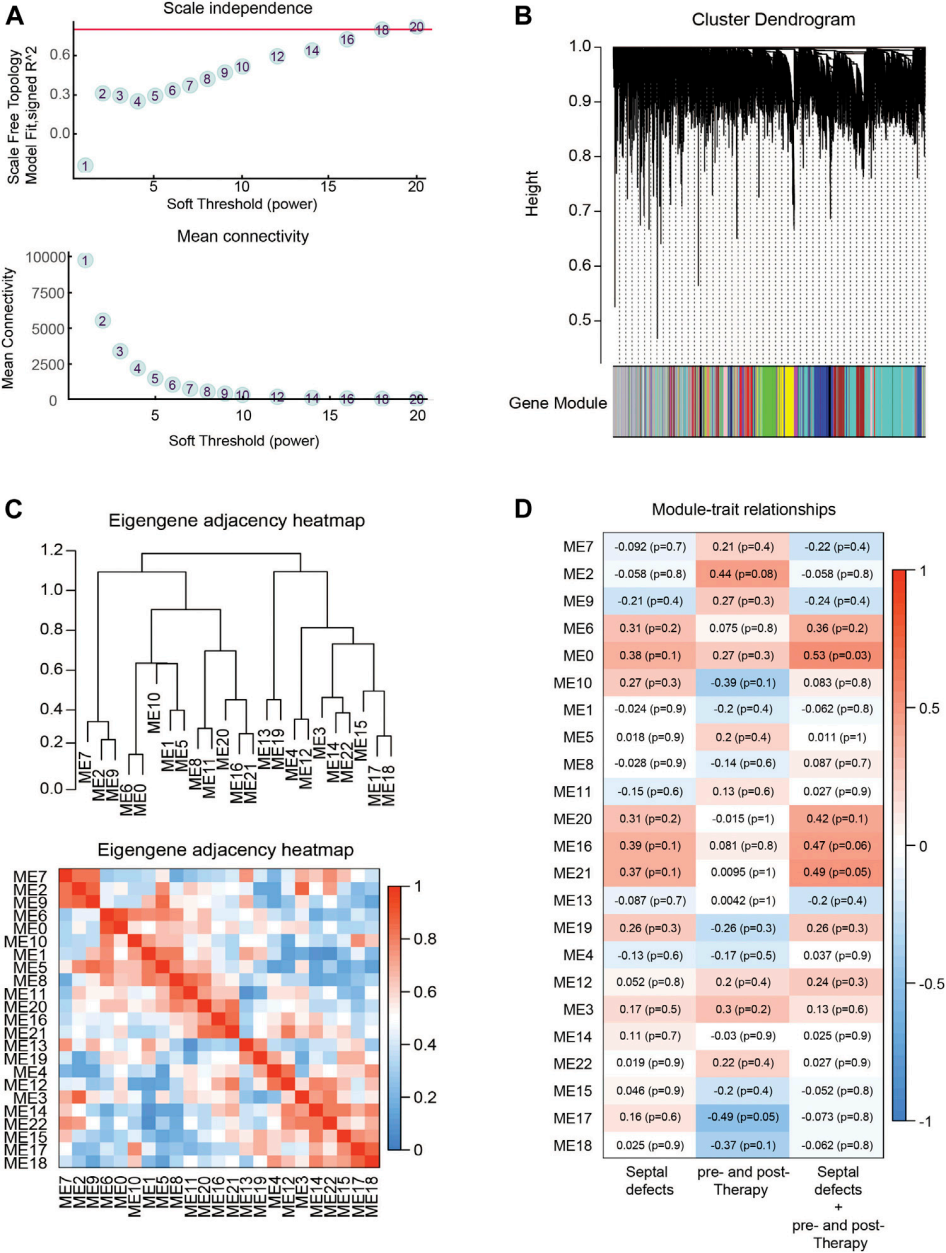
Identification of key modules through WGCNA. **(A)** Analysis of the scale-free fit index **(up)** and the mean connectivity **(down)** for various soft-thresholding powers. **(B)** Eigengenes adjacency heatmap. **(C)** Dendrogram of all DEGs clustered based on a dissimilarity measure (1-TOM). **(D)** Heatmap of the correlation between module eigengenes (ME) and traits of septal defects or therapy. Each grid of the heatmap contains the correlation coefficient and *p*-value.

To investigate the important role of gene interactions in ASDs, the weighted gene co-expression network analysis was used to construct an interaction network with genes, in which the nodes represent the genes and the edges depict their associations, the genes having expression commonality are in the same gene network, and the co-expression relationship between genes is generally measured by the expression correlation coefficient between them. By setting soft-thresholding power as 18 (scale free *R* ^2^ = 0.85) and cut height as 0.25, we eventually identified 18 modules (Figures 4A–D; non-clustering DEGs shown in gray). From the heatmap of module–trait correlations, we identified that the M2 was the most highly correlated with therapy of septal defects. In addition, Module annotation by KEGG pathway is shown in Supplementary Table S5.

### Differentially Expressed Genes Were Validated by Quantitative Reverse Transcriptase-Polymerase Chain Reaction

**FIGURE 5:**
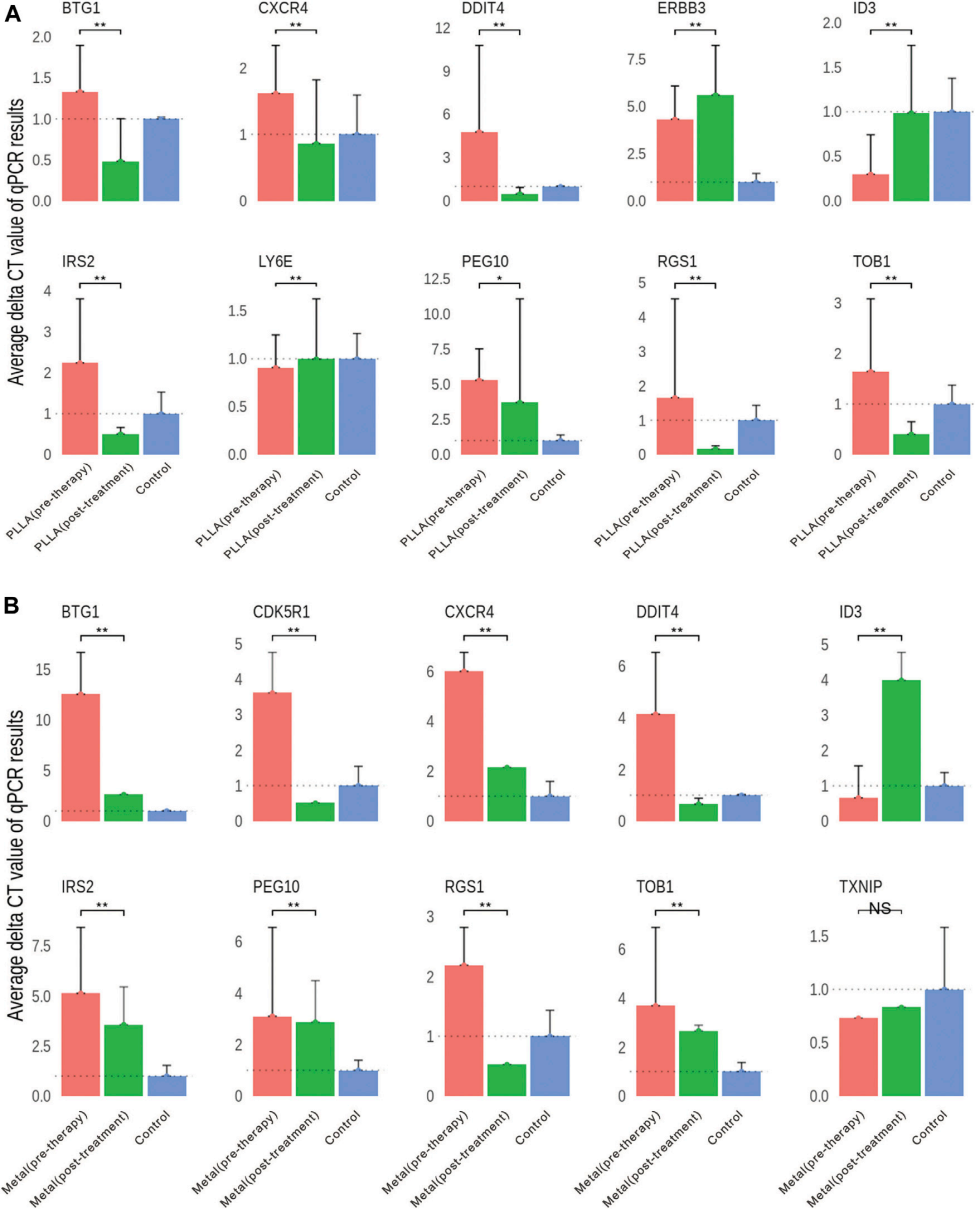
Validation of DEGs by qRT-PCR. **(A)** Gene expression differences in the PLLA device pre-therapy group and post-treatment group. **(B)** Gene expression differences in the metal device pre-therapy group and post-treatment group. NS, no significant; *p < 0.05, **p < 0.01.

Of the total 1,045 genes and 1,523 genes induced by metal and PLLA device treatment, respectively, which were differently expressed in the RNA-seq, down- and upregulated genes with relevance to occlusion device treatment differentially expressed below the *p* < 0.01 and with a logFC >2 were selected for further validation by quantitative RT-PCR. Downregulated genes in both metal and poly-L-lactic acid device treatment (*DDIT4*, *BTG1*, *CXCR4*, *IRS2*, *RGS1*, *PEG10*, and *TOB1*), upregulated genes in poly-L-lactic acid device treatment (*LY6E* and *ERBB3*), and downregulated genes in metal device treatment (*CDK5R1* and *TXNIP*). In addition, the upregulated gene (*ID3*) in both PLLA and metal device treatment was also selected. The results of qRT-PCR are shown in Figure 5. As expected, the expression levels of *DDIT4*, *IRS2*, *TOB1*, *BTG1*, *PEG10*, *CXCR4*, and *RGS1* in both poly-L-lactic acid and metal device treatment were significantly downregulated, which was consistent with a significant decrease in the expression of these genes in the DESeq differential expression analysis. The upregulated *ID3* gene in the DESeq2 differential expression analysis was significantly upregulated by qRT-PCR validated in PLLA and metal device treatment. *LY6E* and *ERBB3* in PLLA device treatment showed upregulation by qRT-PCR and *CDK5R1* in metal device treatment showed downregulation by qRT-PCR validation. However, there were some exceptions to some gene expression; *TXNIP* in metal device treatment was not significantly changed by qRT-PCR validation (Figures 5A,B). The inconsistency between qRT-PCR validation and DESeq2 differential expression analysis may be accounted from varying mRNA levels of the gene in different patient samples.

## Discussion

With the advancement of interventional therapy for congenital heart disease and the progress of device research and development, more and more patients with ASD receive interventional therapy, in which metal and degradable occluders are the two kinds of the most used closure devices in these days (Morray, 2019; Shi et al., 2019; O’Byrne and Levi, 2019; Alnasser et al., 2018). One of the most important indicators for evaluating the histocompatibility of the occluder is the endothelialization induced by the occluders. Endothelialization is crucial and of major clinical importance and impaired endothelialization may lead to prolonged anticoagulant therapy and even serious complications such as residual shunt, device-related thrombosis, endocarditis, and occluder displacement (Chessa et al., 2004; Nguyen et al., 2016; Kalayc and Kalayc, 2017; Chen et al., 2018; Li et al., 2020). Therefore, the observation of endothelialization after occluder implantation is particularly important. However, due to the characteristics that the occluder cannot be removed after implantation *in vivo*, whether the degradable occluders are comparable with the metal occluder in endothelialization is critical. The evaluation of endothelialization is mostly based on the data obtained from animal experiments or a few cases of surgery and autopsy. The observation methods are also limited to electron microscopy, histopathology, and immunohistochemistry (Kuhn et al., 1996; Zahn et al., 2001; Foth et al., 2009; Morray, 2019; Shi et al., 2019).

Few reports have evaluated the process of occluder endothelialization in human by observing the differential expression of genes by RNA-sequencing technology, as well as studies on the mechanism of gene regulation of this process. Therefore, in this study, we carried out RNA-sequencing technology combined with qRT-PCR validation to determine the DEGs and its function on biological processes in the occlusion device (metal or PLLA device) treatment. Transcriptome profile revealed that a total of 1,045 and 1,523 confidently detected genes, respectively, are differentially expressed (FDR < 0.05), of which 337 genes were downregulated and 668 genes were upregulated in metal device post-treatment, and 737 genes were downregulated and 786 genes were upregulated in PLLA device post-treatment. GO analysis revealed the enrichment of these DEGs on the biological process. Then, the differential expression of RNA-Seq data was verified by qRT-PCR, and this differential expression finding confirmed that occluder implantation produced a series of molecular biological changes at the level of gene regulation in the human body, which was finally manifested as endothelialization on the device surface. Theoretically, by observing the differential expression of RNA-Seq data at different time points in the same individual after occluder implantation, it can reflect the degree of endothelialization on the surface of the occluder, making it possible to monitor the endothelialization induced by occluder *in vivo* by RNA-sequencing technology, and also providing a basis for further study of the specific mechanism of gene-level regulation of endothelialization after occluder implantation in patients.

Since the closure of different materials at the same site may involve many similar gene regulatory mechanisms, there are too many overlapping genes, and it is difficult to highlight the genes that play the most critical regulatory role. Therefore, in this study, different material occluders were selected to occlude ASDs, that is, biodegradable or metal materials to occlude ASDs, hoping to select genes that play a key regulatory role among the overlapping expressed genes by RNA-sequencing technology. According to many previous studies observing the process of occluder endothelial coverage, it has been confirmed that the process of endothelialization is similar to wound healing and is a complex biological process of tissue repair (Lock et al., 1989; Sideris et al., 1990; Das et al., 1993; Kuhn et al., 1996; Sharafuddin et al., 1997; Thomsen et al., 1998; Zahn et al., 2001). These include fibroblasts embedded in loose collagen extracellular matrix, newly formed blood vessels, and inflammatory cells (Reinke and Sorg, 2012; Sinno and Prakash, 2013). Degradable occluders differ from metal occluders in structure, require different endothelialization time, but have similar pathophysiological changes, and neo-endothelialization, angiogenesis, and extracellular matrix accumulation are the key events to control the process. Therefore, it is reasonable to believe that in the overlapping part of gene expression between degradable and metal occluders, genes that play a role in regulating cytokines related to neo-endothelialization, angiogenesis, or extracellular matrix accumulation are key regulatory genes.

Our results showed that *CXCR4*, *DDIT4*, and *TOB1* were the highest before occluder treatment and downregulated after treatment with both PLLA and metal device. Previous studies demonstrated that *DDIT4* regulates cell growth, proliferation, and survival by inhibiting the activity of mammalian mTORC1 targets (Wang et al., 2015), while *TOB1*, as an anti-proliferative gene, can regulate cell growth and differentiation and has a migratory role (Liu et al., 2015; Guan et al., 2017; Shangguan et al., 2019). Finally, we also confirmed that CXCR4 is a candidate gene responsible for cardiac congenital pathologies in human as previously suggested in mouse studies (Escot et al., 2013; Wang et al., 2014; Zhong and Rajagopalan, 2015; Page et al., 2018).

Immune response genes and pathways (Supplementary Table S2, Figure 3C) were also identified in our study. Similar clinical studies in device closures of ASDs in children also found that systemic inflammatory reactions occurred after device closure of ASDs in pediatric patients. However, these inflammatory reactions were more significant in patients who underwent a transthoracic approach than in patients who underwent a transcatheter approach (Hong et al., 2020).

Several studies showed that ribosomal protein mutations are associated with patients in Diamond–Blackfan anemia patients with septal defects (Gazda et al., 2008; Chae et al., 2014). Our GO and PPI analysis also provided support for these findings (Figure 3D).

Therefore, it can be preliminarily speculated that *CXCR4*, *DDIT4*, and *TOB1* may be key regulatory genes in the process of endothelialization, and the process of endothelialization may be promoted by downregulation of *CXCR4*, *DDIT4*, and *TOB1* expression after occluder implantation. The differential changes of *CXCR4*, *DDIT4*, and *TOB1* before and after closure also provide a direction for further establishment of knockout model studies to verify the key regulatory genes of endothelialization after implantation.

## Conclusion

In this study, we analyzed RNA-Seq data from the PLLA device therapy group, metal device treatment group, and healthy volunteer group. We found potential genes and pathways that may be involved in endothelialization and remodeling in the progress of atrial septal defect repair, making it possible to monitor the endothelialization of occluders *in vivo* by RNA-seq and RT-PCR methods. At the same time, the changes in gene expression levels and their involvement in different pathways showed that *CXCR4, DDIT4*, and *TOB1* may be key regulatory genes for endothelialization induced by occluder implantation *in vivo*. Our study provides a basis for further research on the underlying mechanisms of regulation endothelialization progression at the transcriptional level after occluder implantation in human.

## Data Availability

The datasets presented in this study can be found in online repositories. The names of the repository/repositories and accession number(s) can be found in the article/Supplementary Material.
